# Striking a new path in reducing cartilage breakdown: combination of antioxidative therapy and chondroanabolic stimulation after blunt cartilage trauma

**DOI:** 10.1111/jcmm.13295

**Published:** 2017-08-22

**Authors:** Jana Riegger, Helga Joos, Hans‐Georg Palm, Benedikt Friemert, Heiko Reichel, Anita Ignatius, Rolf E. Brenner

**Affiliations:** ^1^ Division for Biochemistry of Joint and Connective Tissue Diseases Department of Orthopedics University of Ulm Ulm Germany; ^2^ Department of Orthopedics and Trauma Surgery German Armed Forces Hospital Ulm Ulm Germany; ^3^ Department of Orthopedics University of Ulm Ulm Germany; ^4^ Institute of Orthopedic Research and Biomechanics University of Ulm Ulm Germany

**Keywords:** post‐traumatic osteoarthritis, fibroblast growth factor 18, bone morphogenetic protein 7, insulin‐like growth factor 1, N‐acetyl cysteine, multidirectional therapy

## Abstract

Cartilage injury can trigger crucial pathomechanisms, including excessive cell death and expression of matrix‐destructive enzymes, which contribute to the progression of a post‐traumatic osteoarthritis (PTOA). With the intent to create a novel treatment strategy for alleviating trauma‐induced cartilage damage, we complemented a promising antioxidative approach based on cell and chondroprotective N‐acetyl cysteine (NAC) by chondroanabolic stimulation. Overall, three potential pro‐anabolic growth factors – IGF‐1, BMP7 and FGF18 – were tested comparatively with and without NAC in an *ex vivo* human cartilage trauma‐model. For that purpose, full‐thickness cartilage explants were subjected to a defined impact (0.59 J) and subsequently treated with the substances. Efficacy of the therapeutic approaches was evaluated by cell viability, as well as various catabolic and anabolic biomarkers, representing the present matrix turnover. Although monotherapy with NAC, FGF18 or BMP7 significantly prevented trauma‐induced cell dead and breakdown of type II collagen, combination of NAC and one of the growth factors did not yield significant benefit as compared to NAC alone. IGF‐1, which possessed only moderate cell protective and no chondroprotective qualities after cartilage trauma, even reduced NAC‐mediated cell and chondroprotection. Despite significant promotion of type II collagen expression by IGF‐1 and BMP7, addition of NAC completely suppressed this chondroanabolic effect. All in all, NAC and BMP7 emerged as best combination. As our findings indicate limited benefits of the simultaneous multidirectional therapy, a sequential application might circumvent adverse interferences, such as suppression of type II collagen biosynthesis, which was found to be reversed 7 days after NAC withdrawal.

## Introduction

As the most prevalent joint disease in developed countries, osteoarthritis (OA) has an immense economic and social influence on contemporary society 1. Consequently, there is a growing demand for effective therapies to cure or prevent OA, in particular with respect to the early onset of the so‐called PTOA, mostly affecting the middle‐aged population 2. Due to the complexity of its pathogenesis, the underlying molecular mechanisms are still incompletely understood and pharmacotherapeutic approaches are largely insufficient so far.

Research findings showed that blunt cartilage impact provokes loss of viable cells by acute necrosis and ongoing apoptosis 3, 4. Furthermore, surviving chondrocytes exhibit aberrant overexpression of catabolic collagenases and aggrecanases, such as matrix metalloproteinases (MMPs) and a disintegrin and metalloproteinase with thrombospondin motifs (ADAMTS), causing subsequent degradation of the extracellular matrix (ECM) 5, 6. Simultaneously, biosynthesis of the main ECM components type II collagen and aggrecan 6 diminishes during OA progression, inter alia through chondrocyte desensitization towards insulin‐like growth factor 1 (IGF‐1) 7, 8. The pathologic changes in chondrocyte behaviour are largely triggered by the release of pro‐inflammatory cytokines as well as accumulation of reactive oxygen (ROS) and nitrogen (RNS) species 9, 10. Moreover, availability of bone morphogenetic protein 7 (BMP7), another chondroanabolic growth factor, shows a decline in elderly and arthritic cartilage 11, 12. In conjunction with the decrease in anabolic processes, the prevalent excess of matrix‐degrading proteases provokes a disastrous misbalance, ending up in progressive cartilage degeneration 13.

Previously, we implemented an antioxidative therapy approach based on NAC, which exhibits potent cell and chondroprotective features after *ex vivo* cartilage trauma 14, 15. The neutralization of harmful ROS/RNS by NAC protects chondrocytes from apoptosis and attenuates activation of redox‐sensitive signalling pathways, responsible for catabolic gene expression. Apart from this, NAC‐mediated suppression of COL2A1 gene expression indicated an adverse impact on cartilage anabolism 14. In the following study, we tried to countervail this potential drawback, by parallel stimulation with a pro‐anabolic growth factor. To sustain or restore the chondrogenic potential of the surviving chondrocytes, we compared three pro‐anabolic growth factors, commonly discussed as promising candidates for cartilage regeneration: IGF‐1 7, 16, 17, BMP7 18, 19, 20 and fibroblast growth factor 18 (FGF18) 21, 22, 23. All approaches were evaluated by a variety of outcome parameters, referring to central pathogenetic aspects of PTOA: (*i*) cell viability, (*ii*) gene expression of ECM‐destructive enzymes as well as relevant ECM components, (*iii*) levels of anabolic and catabolic biomarkers, representing the matrix turnover 24, and (*iv*) histological tissue characteristics.

To our knowledge, this is not only the first comparative validation regarding the efficacy of the present growth factors in a human *ex vivo* cartilage trauma model, but also the first multidirectional approach in this context. Consequently, this study exhibits an informative overview of the respective advantages and disadvantages and takes first steps on a new path in preventing or delaying the onset of PTOA.

## Materials and methods

### Specimen preparation and cultivation conditions

Human cartilage was obtained from donors undergoing total knee joint replacement due to OA. Informed consent was obtained from all patients according to the terms of the Ethics Committee of the University of Ulm. Overall, macroscopically intact tissue samples (International Cartilage Repair Society score ≤1) 25 from femoral condyles of 24 patients (mean age 70 years, ranging 50–82 years) were included in the study. Full‐thickness cartilage explants (Ø = 6 mm) were harvested, weighed and cultivated in serum‐containing medium for 24 hrs in an incubator (37°C, 5% CO_2_, 95% humidity). Afterwards, the explants were traumatized and cultivated up to 14 days in serum‐free medium (media composition: S1 in Data S1).

### Ethics

This study was performed in accordance with the Declaration of Helsinki and the guidelines of the Ethical Committee of the University of Ulm.

### Impact loading and subsequent treatment

Cartilage explants were subjected to a single impact load of 0.59 J (approximately 0.2 sec.) using a drop‐tower model as previously described 14, 26. Unloaded explants served as controls. Impacted/unimpacted cartilage explants were incubated w/and w/o therapeutic additives: a growth factor (100 ng/ml rhIGF‐1, 200 ng/ml rhFGF18 or 100 ng/ml rhBMP7; Peprotech, Hamburg, Germany) w/or w/o N‐acetyl‐L‐cysteine (2 mM or 3.5 mM NAC, Sigma‐Aldrich, Taufkirchen, Germany). Fresh additives were added concomitantly with medium change every 2–3 days.

### mRNA isolation and cDNA synthesis

For total RNA isolation, cryopreserved cartilage explants were pulverized with a microdismembrator S (B. Braun Biotech, Melsungen, Germany). Subsequently, RNA was isolated using the Lipid Tissue Mini Kit (Qiagen, Hilden, Germany). RNA was reverse transcribed with the Omniscript RT Kit (Qiagen) and used for quantitative real‐time PCR analysis (StepOnePlus™ Real‐Time PCR System, Applied Biosystems, Darmstadt, Germany).

### Quantitative real‐time polymerase chain reaction (qRT‐PCR)

Determination of the relative expression levels was performed by means of qRT‐PCR analysis (2^−ΔΔCt^ method). To detect desired sequences, TaqMan^®^ Gene Expression Master Mix for TaqMan^®^ Gene Expression Assay (both Applied Biosystems) was used for following probes: Hs00153936_m1 (ACAN), Hs00192708_m1 (ADAMTS4), Hs00199841_m1 (ADAMTS5), Hs01034913_g1 (BMPR1A), Hs00176148_m1 (BMPR2), Hs00164004_m1 (COL1A1), Hs00264051_m1 (COL2A1), Hs00166657_m1 (COL10A1), Hs00179829_m1 (FGFR3), Hs02800695_m1 (HPRT1), Hs00609566_m1 (IGF1R), Hs00899658 (MMP‐1), Hs01548727_m1 (MMP‐2), Hs00968305_m1 (MMP‐3), Hs00233992_m1 (MMP‐13). Power SYBR^®^ Green PCR Master Mix (Applied Biosystems) was used for 18S rRNA, 5′‐CGCAGCTAGGAATAATGGAATAGG‐3′ (forward) and 5′‐CATGGCCTCAGTTCCGAAA‐3′ (reverse), and Platinum^®^ SYBR^®^ Green qPCR SuperMix‐UDG (Invitrogen, Darmstadt, Germany) for GAPDH, 5′‐TGGTATCGTGGAAGGACTCATG‐3′ (forward) and 5′‐TCTTCTGGGTGGCAGTGATG‐3′ (reverse). mRNA expression was determined by normalizing the expression levels separately to the endogenous controls (18S rRNA, GAPDH and HPRT1), and subsequently calculating the ratio mean values in relation to the gene expression level of the untreated, unimpacted control.

### Live/dead cell cytotoxicity assay

To determine the percentage of viable cells, a Live/Dead^®^ Viability/Cytotoxicity Assay (Molecular Probes, Invitrogen) was performed. Unfixed tissue sections (0.5 mm thickness) were stained with 1 μM calcein AM and 2 μM ethidium homodimer‐1 for 30 min. After washing in PBS, they were microscopically analysed by means of a z‐stack module (software AxioVision, Carl Zeiss, Jena, Germany) (S2 in Data S1).

### Analysis of culture media

Quantity of biomarker release into culture media was evaluated by means of enzyme‐linked immunosorbent assays (ELISAs): secreted MMP‐13 was determined using the Human Quantikine ELISA kit (RayBiotech, Norcross, GA, USA). Evaluation of type II collagen synthesis was performed using a CPII ELISA (Ibex, Quebec, Canada). The assay quantifies type II collagen carboxy propeptide (CP II) cleaved from procollagen II after its release into the matrix and directly correlates with newly synthetized type II collagen. Degradation of type II collagen was measured using a C2C ELISA (Ibex), detecting a neoepitope generated during collagenase‐mediated breakdown of type II collagen. The total amount of MMP‐13, C2C and CP II, respectively, was relativized on the weight multiplied by cell viability of the corresponding cartilage explant 14.

### Histological and immunohistochemical analysis

Staining methods are briefly described in the (S5 in Data S1).

### Statistical analysis

Experiments were analysed using GraphPad Prism version 6.0 h (GraphPad Software). Data sets with *n* ≥ 5 were tested for outliers with the Grubbs outlier test. Outliers were not included in statistical analyses. For parametric data sets, a one‐way analysis of variance (anova) with Bonferroni post‐test was used. Nonparametric data sets were analysed by a Kruskal–Wallis test with Dunn′s post‐test. Significant level was set to α = 0.05. Values in diagrams are given as boxplots (median; whiskers: min to max).

## Results

### BMP7 and IGF‐1, but not FGF18, exhibit chondroanabolic qualities referring to anabolic gene expression

To ensure the chondrocytes responsiveness to the applied growth factor concentrations, effects of IGF‐1, FGF18 or BMP7 on gene expression pattern of impacted and unimpacted cartilage explants, respectively, were evaluated first (Table 1).

**Table 1:** Effects of growth factors on anabolic and catabolic gene expression pattern of human cartilage explants after 7 days

	IGF‐1 (*n* ≥ 8)				FGF18 (*n* ≥ 6)				BMP7 (*n* ≥ 8)			
	[C +GF *versus* C]		[T +GF *versus* T]		[C +GF *versus* C]		[T +GF *versus* T]		[C +GF *versus* C]		[T +GF *versus* T]	
Target gene	Fold change*	*P*‐value	Fold change	*P*‐value	Fold change	*P*‐value	Fold change	*P*‐value	Fold change	*P*‐value	Fold change	*P*‐value
ADAMTS4	4.0	0.5487	−1.7	>0.9999	*16 .0*	<0.0001	1.2	0.8642	1.7	>0.9999	−1.9	>0.9999
ADAMTS5	1.8	0.4953	−1.5	0.4488	*6.2*	<0.0001	*1.9*	0.0073	*2.7*	0.0143	−1.1	>0.9999
MMP‐1	*2.3*	0.0067	1.1	>0.9999	*3.7*	<0.0001	−1.3	>0.9999	1.6	0.4991	1.2	0.9935
MMP‐2	3.8	0.0741	−1.7	0.1771	*8.0*	<0.0001	−*4.3*	0.0125	1.9	>0.9999	−1.6	0.2522
MMP‐3	1.2	>0.9999	1.0	>0.9999	*3.4*	<0.0001	1.3	0.5551	1.2	>0.9999	1.1	>0.9999
MMP‐13	2.6	0.0526	1.2	0.4446	*18.5*	0.0007	1.8	0.1002	2.3	>0.9999	*−*1.6	0.3856
COL1A1	8.0	0.1302	*−5.5*	0.0247	*16.7*	0.0008	−*18.1*	0.0247	5.9	0.14	*−8.6*	0.0218
COL2A1	*2.6*	<0.0001	*1.5*	0.0069	*−2.8*	0.0308	*−3.9*	0.0003	*1.5*	0.0283	*2.1*	<0.0001
COL10A1	*3.9*	0.0489	*−*1.8	0.6805	1.9	>0.9999	*−*1.3	>0.9999	1.2	>0.9999	1.2	>0.9999
ACAN	1.2	0.2004	*−*1.1	>0.9999	*−2.9*	<0.0001	*−2.9*	<0.0001	*1.3*	0.0375	*1.4*	0.0017

C= untreated, unimpacted control, GF= growth factor, T= traumatized, T+GF= traumatized and stimulated with growth factor. *Significant differences between groups (fold change) were italicized.

After stimulation with IGF‐1, unimpacted cartilage explants showed enhanced gene expression levels of catabolic enzymes ADAMTS‐4 (*P* = 0.5487), MMP‐1 (*P* = 0.0067), MMP‐2 (*P* = 0.0741) and MMP‐13 (*P* = 0.0526). Likewise, IGF‐1 stimulation significantly enhanced the gene expression of COL2A1 and COL10A1 (*P* < 0.0001 and *P* = 0.0489, respectively) as well as COL1A1 by trend. In impacted cartilage explants, gene expression of COL2A1 (*P* = 0.0069) was significantly increased after IGF‐1 stimulation, while that of COL1A1 (*P* = 0.0247) was suppressed.

Stimulation of unimpacted cartilage explants with FGF18 showed significant induction of catabolic gene expression in case of ADAMTS‐4 (*P* < 0.0001), ADAMTS‐5 (*P* < 0.0001), MMP‐1 (*P* < 0.0001), MMP‐2 (*P* < 0.0001), MMP‐3 (*P* < 0.0001) and MMP‐13 (*P* = 0.0007), as well as COL1A1 (*P* = 0.0008). Simultaneously, FGF18 stimulation significantly suppressed the expression of ACAN (*P* < 0.0001) and COL2A1 (*P* = 0.0308). In impacted cartilage explants, FGF18 stimulation significantly suppressed trauma‐induced expression of MMP2 (*P* = 0.0125) and COL1A1 (*P* = 0.0247) but also ACAN (*P* < 0.0001) and COL2A1 (*P* = 0.0003).

In unimpacted cartilage explants, BMP7 stimulation significantly increased the expression of COL2A1 (*P* = 0.0283) and ACAN (*P* = 0.0375) but also ADAMTS5 (*P* = 0.0143) as well as COL1A1 by trend. As compared to non‐stimulated impacted cartilage explants, BMP7 treatment significantly enhanced levels of COL2A1 (*P* < 0.0001) and ACAN (*P* = 0.0017). As stimulation with growth factors revealed largely significant influence on the expression of the tested target genes, some efficacy has been considered.

### Multidirectional antioxidative and chondroanabolic therapy of impacted cartilage tissue shows no additional benefit as compared to the mono‐therapeutic approaches

Seven days after trauma (T), gene expression of all tested ECM‐destructive proteases was significantly elevated as compared to control level (C): ADAMTS‐4 (9.2‐fold, *P* < 0.0001), ADAMTS‐5 (threefold, *P* = 0.0032), MMP‐1 (11.5‐fold, *P* < 0.0001), MMP‐2 (fourfold, *P* < 0.0001), MMP‐3 (2.3‐fold, *P* < 0.0001) and MMP‐13 (10.5‐fold *P* < 0.0001). While the expression of COL2A1 and ACAN was rather down‐regulated, that of COL1A1 was significantly enhanced after trauma (25.7‐fold, *P* = 0.0005). Moreover, trauma induced the gene expression of COL10A1 by trend (2.9‐fold).

**Figure 1:**
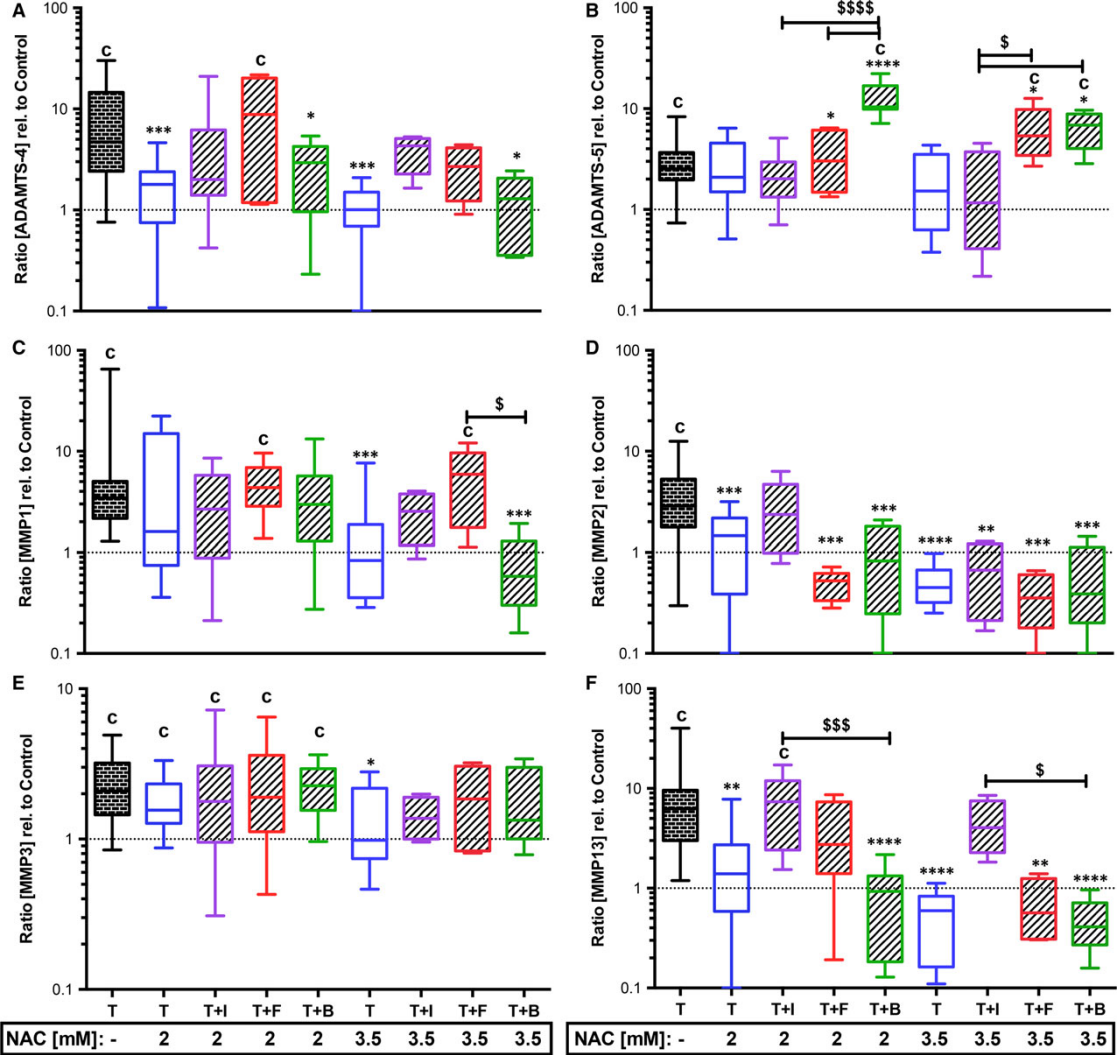
Effects of growth factors with NAC on trauma‐induced gene expression of ECM‐destructive enzymes. Impacted human cartilage explants were continuously treated by growth factors and NAC (2 mM or 3.5 mM). 7 days post‐trauma, gene expression levels of ECM‐destructive enzymes (**A**) ADAMTS‐4, (**B**) ADAMTS‐5, (**C**) MMP‐1, (**D**) MMP‐2, (**E**) MMP‐3 and (**F**) MMP‐13 were analysed by qRT‐PCR. T= traumatized, +I/B/F= stimulated by IGF‐1 (purple)/FGF18 (red)/BMP7 (green). Significant differences between groups were depicted as: [*versus* T] *P < 0.05, **P < 0.01, ***P < 0.001; ****P < 0.0001; [*versus* C] c= *P* < 0.01; [GF versus GF] ^$^ *P* < 0.05, ^$$$^ *P* < 0.001, ^$$$$^ *P* < 0.0001. T: *n* ≥ 20; T+N: *n* ≥ 13 (2 mM), *n* ≥ 9 (3.5); T+N+I: *n* ≥ 8 (2 mM), *n* = 4 (3.5 mM); T+N+F: *n* ≥ 5 (2 mM), *n* = 5 (3.5 mM); T+N+B: *n* ≥ 8 (2 mM), *n* = 5 (3.5 mM).

**Figure 2:**
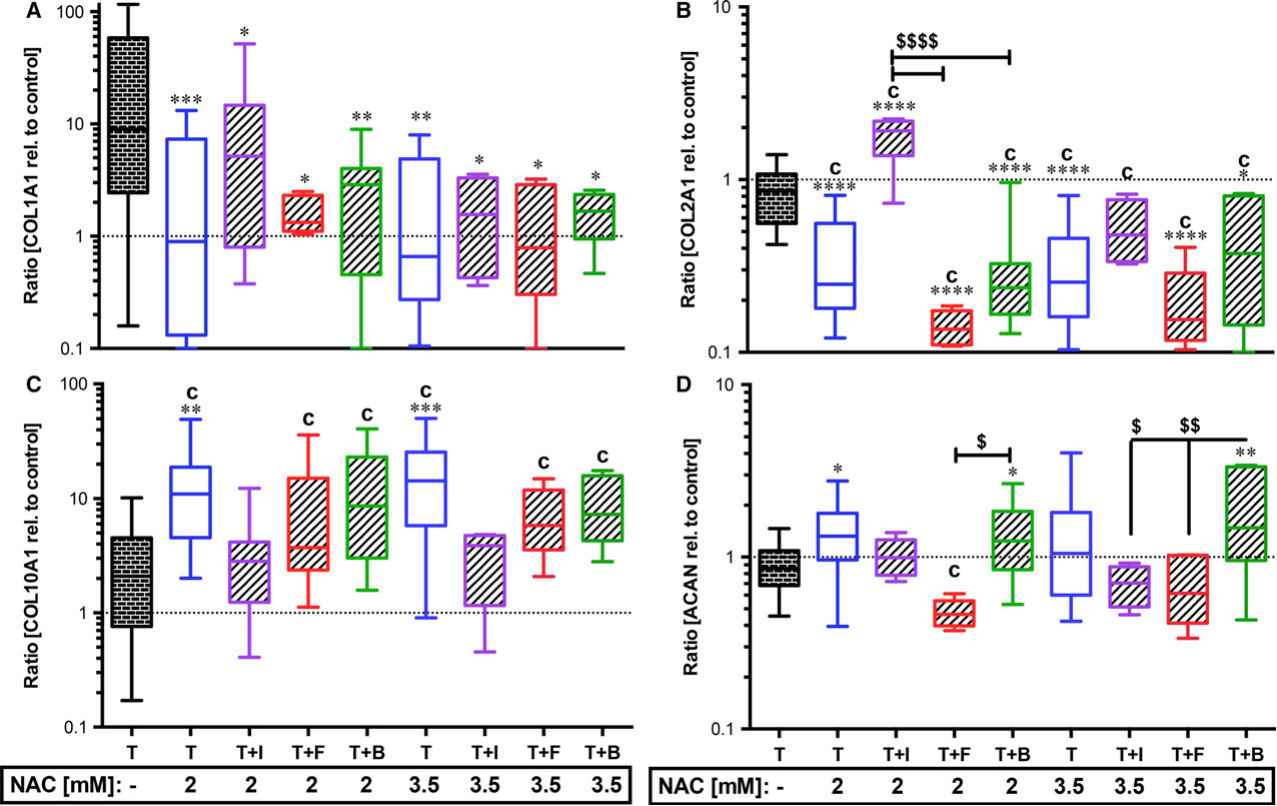
Effects of growth factors with NAC on trauma‐related alterations in gene expression of ECM components. Impacted human cartilage explants were continuously treated by growth factors and NAC (2 mM or 3.5 mM). 7 days post‐trauma, gene expression levels of ECM components (**A**) type I collagen (COL1A1), (**B**) type II collagen (COL2A1), (**C**) type X collagen (COL10A1) and (**D**) aggrecan (ACAN) were analysed by qRT‐PCR. T= traumatized, +I/B/F= stimulated by IGF‐1 (purple)/FGF18 (red)/BMP7 (green). Significant differences between groups were depicted as: [*versus* T] *P < 0.05, **P < 0.01, ***P < 0.001; ****P < 0.0001; [*versus* C] c= *P* < 0.05; [GF *versus* GF] ^$^ *P* < 0.05, ^$$^P < 0.01, ^$$$$^ *P* < 0.0001. T: *n* ≥ 20; T+N: *n* ≥ 13 (2 mM), *n* ≥ 9 (3.5); T+N+I: *n* ≥ 13 (2 mM), *n* = 4 (3.5 mM); T+N+F: *n* ≥ 6 (2 mM), *n* = 5 (3.5 mM); T+N+B: *n* ≥ 8 (2 mM), *n* = 5 (3.5 mM).

Additional IGF‐1 stimulation largely reversed favourable effects of NAC treatment on expression of ACAN (Fig. 2D), ADAMTS‐4, MMP‐2 and MMP‐13 (Fig. 1A, D and F); however, MMP‐2 gene expression was significantly suppressed using a higher NAC concentration (−5.7‐fold, *P* = 0.0047). NAC‐induced gene expression of COL10A1 was likewise reversed by IGF‐1 (Fig. 2C). Although 2 mM NAC treatment usually caused significant suppression of COL2A1 (Fig. 2B), it was significantly enhanced in combination with IGF‐1 (twofold, *P* < 0.0001). This effect was eliminated by the use of the higher NAC concentration.

Combined treatment with FGF18 and NAC significantly suppressed the trauma‐induced expression of MMP‐2 ([2 mM] −8.2‐fold, *P* = 0.0006; [3.5 mM] −10.4‐fold, *P* = 0.0004; Fig. 1D) and MMP‐13 ([3.5 mM] −14.8‐fold, *P* = 0.0045; Fig. 1F), but also ACAN ([2 mM] *P* = 0.05, relative to C; Fig. 2D) and COL2A1 ([2 mM] and [3.5 mM] *versus* C and T: *P* < 0.0001; Fig. 2B). Addition of NAC, completely abolished the inducing effect of BMP7 on COL2A1 ([2 mM] *versus* C and T: *P* < 0.0001, [3.5 mM] *versus* T: *P* = 0.0188; *versus* C: *P* < 0.0001, Fig. 2B), whereas ACAN (Fig. 2D) was further enhanced, indicating an additive effect of the two therapeutics ([2 mM] 1.5‐fold, *P* = 0.0206; [3.5 mM] 2.1‐fold, *P* = 0.007). Except for ADAMTS‐5 and MMP‐3 (Fig. 1B and E), NAC‐mediated suppression of trauma‐induced catabolic gene expression was broadly maintained, but not significantly enhanced in combination with BMP7: [2 mM] ADAMTS‐4: −3.5‐fold, *P* = 0.0137; MMP‐2: −3.8‐fold, *P* = 0.0004; MMP‐13: −11.9‐fold, *P* < 0.0001; [3.5 mM] ADAMTS‐4: −7.3‐fold, *P* = 0.0134; MMP‐1: −14.7‐fold, *P* = 0.0017; MMP‐2: −7‐fold, *P* = 0.0001; MMP‐13: −21.9‐fold, *P* < 0.0001.

Elevated COL10A1 gene expression after NAC treatment tended to be lowered in combination with growth factor stimulation, but was still significantly increased with FGF18 (*versus* C: [2 mM] *P* = 0.0096, [3.5 mM]: *P* = 0.0185) or BMP7 (*versus* C: [2 mM] *P* = 0.0004, [3.5 mM]: *P* = 0.0016). Moreover, trauma‐induced gene expression of ADAMTS‐5 (Fig. 1B) was significantly enhanced by the combined treatment using NAC and FGF18 ([3.5 mM] *P* = 0.05) or BMP7 ([2 mM] *P* < 0.0001, [3.5 mM]: *P* = 0.0135).

After all, NAC‐mediated induction of COL10A1 and suppression of COL2A1 gene expression, respectively, were completely eliminated 7 days after deprivation of NAC application (Fig. S3A and C in Data S1).

Additionally, gene expression levels of corresponding growth factor receptors BMP receptor 1A (BMPR1A, Fig. S4A in Data S1) and 2 (BMPR2, Fig. S4B in Data S1), IGF‐1 receptor (IGF1R, Fig. S4C in Data S1) and FGF receptor 3 (FGFR3, Fig. S4D in Data S1) were exemplarily evaluated. After trauma, gene expression levels of IGF1R and FGFR3 were enhanced by trend. mRNA levels of IGFR1 were significantly induced by BMP7 stimulation (*versus* C [C+B] *P* = 0.0167). While both BMPR1A and BMPR2 were significantly enhanced by combined treatment with BMP7 and NAC (*versus* C: *P* = 0.0263 and *P* < 0.0001, respectively), FGFR3 was significantly attenuated after FGF18 stimulation in all tested approaches (*versus* C: [C+F] *P* = 0.0018; [T+F] *P* = 0.0463; [T+F+N] *P* = 0.0364).

### FGF18 and BMP7 exhibit cell protective qualities after cartilage trauma, whereas IGF‐1 only shows low efficiency

**Figure 3:**
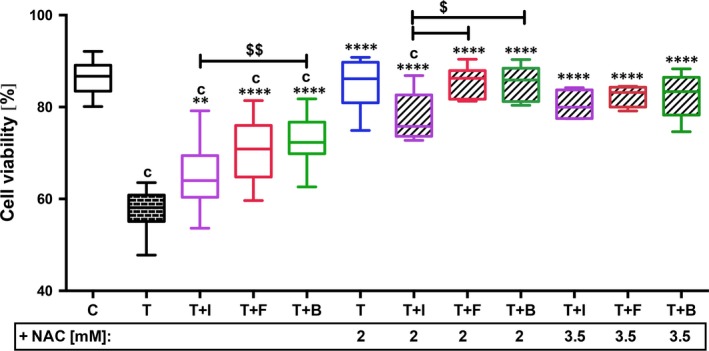
Effects of growth factors w/and w/o NAC on cell viability after cartilage trauma. Impacted human cartilage explants were continuously treated by GF w/and w/o NAC ([2 mM]: *n* ≥ 7; [3.5 mM]: *n* ≥ 4) for 7 days and subsequently analysed by Live/Dead^®^ Viability/Cytotoxicity Assay. C= control, T= traumatized, +I/B/F= stimulated by IGF‐1 (purple)/FGF18 (red)/BMP7 (green). Significant differences between groups were depicted as: [*versus* T] **P < 0.01, ****P < 0.0001; [*versus* C] c= *P* < 0.001; [*versus* T+N] TN= *P* < 0.05; [GF *versus* GF] ^$^ *P* < 0.05, ^$$^ *P* < 0.01.

Cell viability of the cartilage explants was evaluated 7 days post‐trauma (Fig. 3). Traumatization of cartilage explants caused significant decrease in cell viability (−28.6%, *P* < 0.0001). Although stimulation with growth factors significantly increased cell viability of impacted cartilage explants (T+IGF: +5.9%, *P* = 0.007; T+FGF: +12.8%, *P* < 0.0001; T+BMP: +14.4%, *P* < 0.0001), it was still significantly lower than C. IGF‐1 had an inferior efficacy as compared to FGF18 (−5.7%, *P* = 0.3078) and BMP7 (−8%, *P* = 0.0098). The same could be observed in the combined approach with 2 mM NAC: cell viability of T+N+IGF was still significantly lower as C (−11%, *P* = 0.0013), T+N+FGF (−10.5%, *P* = 0.0463) and T+N+BMP (−10%, *P* = 0.0411). Nevertheless, cell viability of impacted cartilage explants simultaneously treated with 2 mM NAC and FGF18 or BMP7, respectively, was equal to that of C or T+N (mean difference to C ranging 0.7127–1.373). Even in combination with 3.5 mM NAC (exemplarily tested), IGF‐1 stimulation could not achieve full recovery of the cell viability (−6.76%). Moreover, the higher NAC concentration was rather disadvantageous in combination with FGF18 and BMP7.

### FGF18 and BMP7 show significant chondroprotective qualities w/and w/o NAC

**Figure 4:**
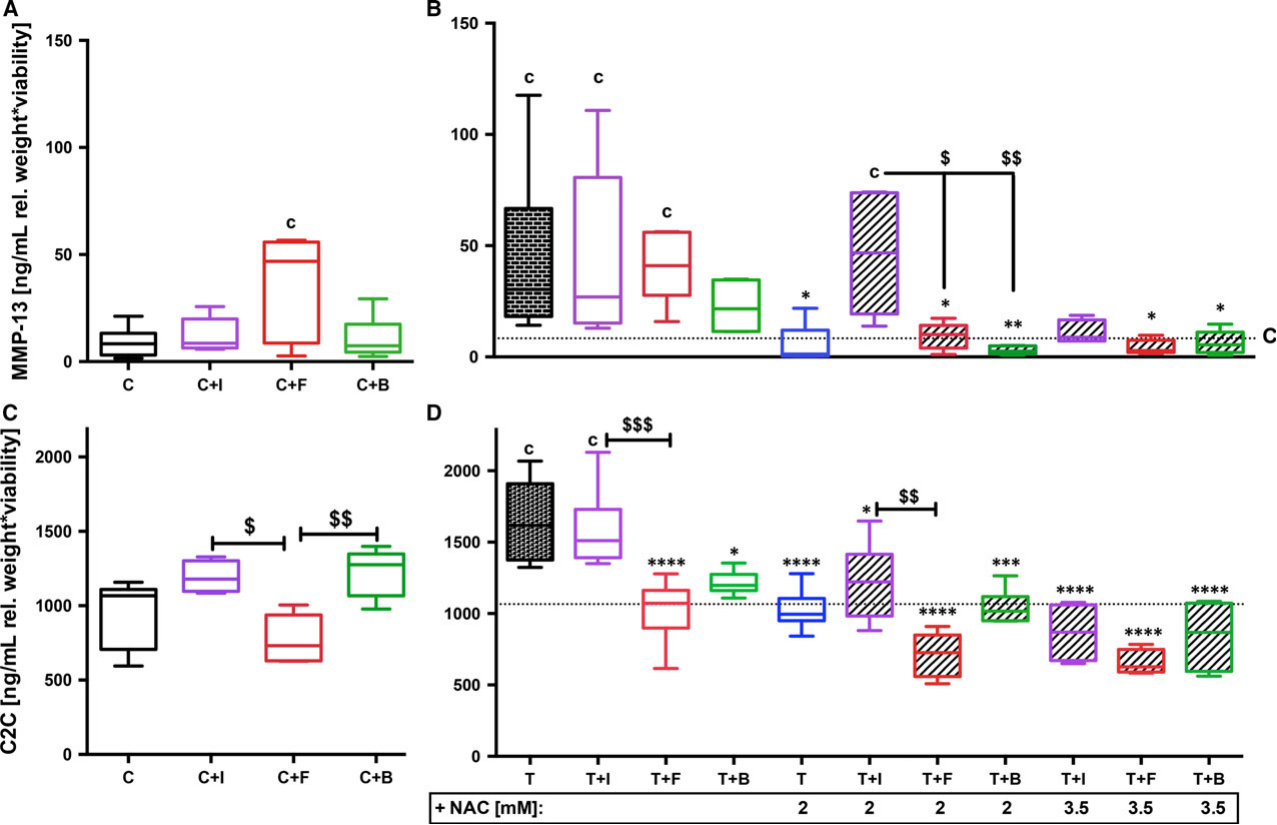
Effects of growth factors w/or w/o NAC on OA‐associated catabolic biomarkers. (**B, D**) Impacted human cartilage explants were continuously treated by GF w/and w/o NAC (2 mM, *n* ≥ 5; or 3.5 mM, *n* ≥ 4). (**A, C**) Corresponding unimpacted controls were stimulated by GF;* n* ≥ 5 each. Amount of (**A, B**) secreted MMP‐13 and (**C, D**) generated type II collagen cleavage product C2C in the culture media was analysed 7 days post‐trauma by means of corresponding ELISAs. C= unimpacted control, T= traumatized, GF= growth factor, +I/B/F= stimulated by IGF‐1 (purple)/FGF18 (red)/BMP7 (green). Significant differences between groups were depicted as: [*versus* T] *P < 0.05, **P < 0.01, ***P < 0.001, ****P < 0.0001; [*versus* C] c= *P* < 0.05; [GF *versus* GF] ^$^ *P* < 0.05, ^$$^ *P* < 0.01, ^$$$^ *P* < 0.001.

The amount of MMP‐13 and the cleavage‐specific neoepitope C2C, generated by collagenases MMP‐1, MMP‐8 and MMP‐13 during breakdown of type II collagen, were used as representative biomarkers of the catabolism. Quantification of secreted MMP‐13 (Fig. 4A and B) widely confirmed the findings of gene expression analysis: MMP‐13 release was significantly increased after FGF18 stimulation of unimpacted cartilage explants (3.7‐fold, *P* = 0.0111). While the trauma‐induced release of MMP‐13 (Fig. 4B) was still significantly enhanced after treatment with either FGF18 (4.7‐fold, *P* = 0.036) or IGF‐1 (4.7‐fold, *P* = 0.014), it was lowered in trend by BMP7 (−2‐fold, *P* = 0.138). Combined treatment of impacted cartilage explants significantly decreased the amount of secreted MMP‐13: T+N+I ([3.5 mM] −4.2‐fold, *P* = 0.0816), T+N+F ([2 mM] −4.8‐fold, *P* = 0.022; [3.5 mM] −10.4‐fold, *P* = 0.0104), T+N+B ([2 mM] −15.4‐fold, *P* = 0.0039; [3.5 mM] −7.2‐fold, *P* = 0.0169). MMP‐13 concentrations were still significantly higher after combined treatment using IGF‐1 and 2 mM NAC as compared to the corresponding treatments using FGF18 (4.9‐fold, *P* = 0.0342) or BMP7 (15.6‐fold, *P* = 0.0072).

Except for C+FGF18, the amount of C2C (Fig. 4C and D) largely correlated with the findings of the secretion of MMP‐13: Trauma‐induced increase of C2C (1.7‐fold, *P* < 0.0001, Fig. 4D) was significantly lowered by FGF18 (−1.6‐fold, *P* < 0.0001) and BMP7 (−1.4‐fold, *P* = 0.0132). In combination with NAC, trauma‐induced breakdown of type II collagen was additionally reduced: T+N+I ([2 mM] −1.3‐fold, *P* = 0.0157; [3.5 mM] −1.9‐fold, *P* < 0.0001), T+N+F ([2 mM] −2.3‐fold; [3.5 mM] −2.5‐fold, both *P* < 0.0001) and T+N+B ([2 mM] −1.6‐fold, *P* = 0.001; [3.5 mM] −2‐fold, *P* < 0.0001). Moreover, FGF18 (w/or w/o 2 mM NAC) had a significant higher effect on prevention of C2C‐release after trauma as compared to corresponding treatments using IGF‐1 ([w/o NAC] *P* = 0.0005; [2 mM] *P* = 0.0018) and even lowered the amount of C2C in the media of unimpacted explants as compared to IGF‐1 (*P* = 0.0126) and BMP7 (*P* = 0.0045).

### IGF‐1 and BMP7 significantly promote type II collagen biosynthesis, whereas their combination with NAC abolishes this effect

**Figure 5:**
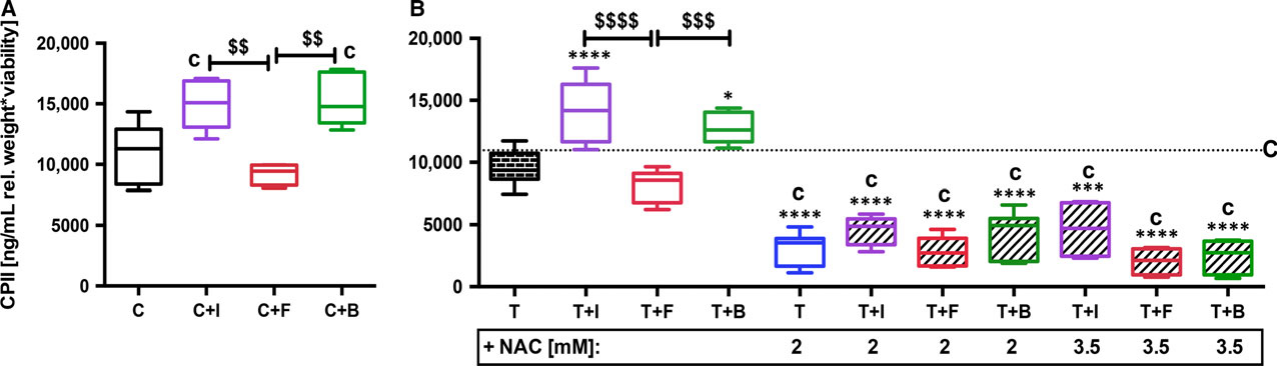
Effects of growth factors w/or w/o NAC on type II collagen synthesis as anabolic biomarker. (**B**) Impacted human cartilage explants were continuously treated by GF w/and w/o NAC (2 mM, *n* ≥ 5; or 3.5 mM, *n* ≥ 4). (**A**) Corresponding unimpacted controls were stimulated by GF;* n* ≥ 5 each. Amount of CPII in the culture media was analysed 7 days post‐trauma by means of ELISA. C= unimpacted control, T= traumatized, GF= growth factor, +I/B/F= stimulated by IGF‐1 (purple)/FGF18 (red)/BMP7 (green). Significant differences between groups were depicted as: [*versus* T] *P < 0.05, ****P < 0.0001; [*versus* C] c= *P* < 0.05; [GF *versus* GF] ^$$^ *P* < 0.01, ^$$$^ *P* < 0.001, ^$$$$^ *P* < 0.0001.

Content of CP II in culture media was considered as an anabolic biomarker and confirmed the findings of gene expression analysis to a great extent: IGF‐1 and BMP7 stimulation significantly supported type II collagen biosynthesis of unimpacted (C+I: 1.38‐fold, *P* = 0.0251; C+B: 1.4‐fold, *P* = 0.0134, Fig. 5A) as well as impacted cartilage explants (T+I: 1.48‐fold, *P* < 0.0001; T+B: 1.33‐fold, *P* = 0.0131, (Fig. 5B). FGF18 showed no inductive but rather suppressive effect on type II collagen biosynthesis, which was significant as compared to IGF‐1 (C+F: −1.6‐fold, *P* = 0.0034; T+F: −1.7‐fold, *P* < 0.0001) or BMP7 (C+F: −1.7‐fold, *P* = 0.002; T+F: −1.6‐fold, *P* = 0.0001). Addition of NAC to any growth factor (Fig. 5B) significantly reduced the amount of CP II in culture media of impacted cartilage explants compared to both T and C (*P* < 0.0001, each).

To test reversibility of type II collagen biosynthesis suppression by NAC, antioxidative therapy was exemplarily deprived after 7 days. Quantification of CP II revealed significant recovery of type II collagen synthesis (*P* = 0.0133) 7 days after deprivation of NAC (14 days post‐trauma), compared to continuously treated impacted cartilage explants (Fig. S3B in Data S1).

### Evaluation of the histomorphologic cartilage quality largely confirmed the previous findings

Histomorphologic cartilage tissue characteristics were evaluated exemplarily in terms of type II collagen and glycosaminoglycan (GAG) content, as well as cell density and distribution. 14 days after trauma, increased numbers of cell clones or clusters as well as column‐like stacking of the cells could be observed with and without growth factor stimulation (Fig. S5A in Data S1). Additional analysis of the cell distribution at an earlier time‐point (7 days post‐trauma) revealed less cell clusters and rather hypocellularity. NAC treatment appeared to lower the trauma‐induced cluster formation after 14 days. Moreover, type II collagen (Fig. S5B in Data S1) and GAG (Fig. S5C in Data S1) staining were less intensive after traumatization as compared to C.

IGF‐1 stimulation of impacted cartilage explants resulted in higher GAG and type II collagen staining intensities. This effect was clearly inhibited in combination with NAC as described before. Surprisingly, NAC did not interfere with chondroanabolic influence of BMP7 and, furthermore, did not attenuate type II collagen staining intensities after mono‐therapeutic application. Once again, no pro‐anabolic influence was found after FGF18 stimulation.

## Discussion

Overall, trauma‐induced pathomechanisms initiate several cellular knock‐on effects, as apoptosis, excessive expression of ECM‐destructive proteases and simultaneous decline of ECM‐synthesis. Additional release of pro‐inflammatory cytokines and accumulation of ROS/RNS, which are crucial key mediators, promote the onset and progression of PTOA 27. Regarding the complexity of PTOA pathogenesis, it would be utopian to search for a single panacea. As recently suggested by Chubinskaya and Wimmer, the ideal therapy should include pro‐anabolic as well as anti‐catabolic features and further support intrinsic regeneration 28. Based on the encouraging results of our recent study 14, a multidirectional therapy, combining the antioxidant NAC and additional chondroanabolic stimulation, seemed to be a promising strategy for approaching the challenging therapeutic requirements to prevent or ameliorate PTOA. Antioxidative treatment by NAC (2 mM or 3.5 mM) has been found to possess significant cell and chondroprotective features after *ex vivo* traumatization of human cartilage 14. NAC not only attenuated trauma‐induced gene expression of the ECM‐destructive enzymes ADAMTS‐4, MMP‐1, MMP‐2, MMP‐3 and MMP‐13, but also the secreted amount and proteolytic activity of MMPs, as shown for MMP‐2 and MMP‐13, respectively. Moreover, NAC administration abolished apoptotic cell death, guaranteeing a significant protection of chondrocyte viability after cartilage injury. Although NAC was found to enhance the gene expression of ACAN, it significantly suppressed that of COL2A1 14. Therefore, the question of possible adverse reaction of NAC treatment concerning the ECM anabolism and maintenance of the chondrogenic phenotype of the surviving cells arouse. With respect to these achievements but also concerns, we assumed that the pro‐anabolic growth factors might complement the ‘deficiencies’ of NAC and suggested a mutual supportiveness between the two therapeutics.

The growth factor concentrations used throughout this study were determined by reliable bibliographic references 18, 21, 29 and in‐house preliminary testing work, including gene expression analysis and chemotaxis assay, respectively (data not shown). Moreover, our current findings exhibited significant influences on mRNA and protein level after growth factor stimulation, which endorsed the efficacy of the applied concentrations. Although there are various studies dealing with these growth factors, most knowledge about their qualities was gained in cell culture 8, 17, 30, cytokine‐induced OA 7, 16 as well as animal *in vitro* 21 and *in vivo* 19, 22 models, respectively. Therefore, our model might add valuable information about the therapeutic potential of growth factors relevance after blunt cartilage trauma (0.59 J) based on a human tissue culture model.

Fourteen days after trauma, increased incidence of cell clusters as well as column‐like cell distribution near the surface and within close proximity to the impact site were observed. Cell columns can usually be found in the deeper zones but are rather uncommon for the upper regions of healthy cartilage 31. Trauma‐induced cell proliferation and subsequent cluster formation may represent an initial attempt of intrinsic regeneration after cartilage injury and has been described as a hallmark of OA cartilage 31. Nevertheless, at the earlier time‐point (7 days), the tissue was rather hypocellular and the cell viability was significantly impaired due to necrotic and apoptotic events 3, 4, 14. Single therapy with IGF‐1, FGF18 or BMP7 for 7 days showed significant but only moderate recovery, amongst which BMP7 exhibited the best results. While proliferative and anti‐apoptotic characteristics have been previously described for IGF‐1 32, 33 and FGF18 21, BMP‐7 is only known for its anti‐apoptotic quality so far 18, 20. Moreover, growth factor stimulation had divergent influence on the gene expression of anabolic and catabolic markers depending on whether the explants were previously impacted. Although IGF‐1, FGF18 and BMP7 tended to induce the gene expression of catabolic enzymes as well as type I collagen in unimpacted control tissue, the induction was attenuated and even mostly reversed in impacted cartilage explants.

In short, FGF18 was the strongest inducer of catabolic gene expression in unimpacted control tissue, whereas BMP7 and IGF‐1 had moderate adverse effects. While BMP7 tended to attenuate both trauma‐induced MMP‐13 secretion and breakdown of type II collagen, IGF‐1 reduced neither of them. Although FGF18 had no attenuating effect on secretion of MMP‐13, significant alleviation of type II collagen cleavage by FGF18 might indicate chondroprotective qualities of the growth factor. Similar prevention of type II collagen breakdown following single impact load was previously observed in a trauma‐model using horse cartilage explants 21. That FGF18 significantly ameliorated collagenase‐mediated cleavage of type II collagen despite enhanced MMP‐13 amounts might correlate with its suppressing effect on MMP‐2 gene expression. Activation of freshly secreted pro‐MMP‐13 is based on a proteolytic cascade, comprising the membrane‐associated MT1‐MMP (MMP‐14) and MMP‐2. Although MMP‐13 can also be directly activated by MT1‐MMP, the reaction is potentiated in the presence of MMP‐2. Further, MMP‐2, which itself is activated by MT1‐MMP, is a much faster activator of MMP‐13 than MT1‐MMP 34.

Unlike IGF‐1 and BMP7, FGF18 had detrimental impact on the expression of ACAN and COL2A1 in both unimpacted and traumatized cartilage explants, which was confirmed on protein level by quantifying the content of CP II in culture media. Although suppression of type II collagen has already been reported in some instances 21, 35, other *in vivo* studies demonstrated promising pro‐anabolic potential of FGF18 22, 23. As currently described, FGF18 significantly down‐regulated type II collagen expression as well as hypertrophy markers and promoted osteogenesis during chondrogenic differentiation of isolated mesenchymal stem cells 36. Further, FGF18 was found to be expressed by hypertrophic chondrocytes adjacent to the tidemark, stimulating terminal differentiation in the osteochondral tissue at the growth plate. It can be assumed that FGF18 is essential for both chondrogenesis and osteogenesis during skeletal development. However, it appears that FGF18 affects chondrogenesis in a negative way, while having a positive impact on osteogenesis 37. Another critical aspect was revealed by gene expression analysis of FGFR3, which was significantly down‐regulated after FGF18 stimulation. While FGF2 is thought to induce catabolic processes by signalling *via* FGFR1, FGF18 exhibits highest receptor specificity to ‘anabolic’ FGFR3 38, 39. According to previous studies, negative regulation of FGFR3 can result from FGF2‐mediated FGFR1‐signalling, whereas no negative feedback of FGF18 on its receptor has been described so far. As it has been shown that the expression ratio of FGFR1 to FGFR3 is highly overbalanced in OA cartilage and FGFR1 expression can be additionally up‐regulated by FGF18, it could be possible that effects of endogenous FGF2 might be potentiated by FGF18 stimulation 39. As we found significant effects of FGF18 despite reduced FGFR3 gene expression, desensitization is rather improbable but might occur in the long run.

Summarizing our results of the chondroanabolic approach, we found that IGF‐1 and BMP7, but not FGF18, possess promising pro‐anabolic qualities after blunt cartilage injury as shown for ACAN and COL2A1 expression as well as type II collagen biosynthesis. Consequently, we identified IGF‐1 and BMP7 as potential candidates to promote the chondroanabolic behaviour in chondrocytes after cartilage trauma. Although FGF18 seemed to be unsuitable to promote anabolic processes, it still exhibited considerable cell‐ and chondroprotective qualities in the context of our human *ex vivo* trauma‐model. This way, it might indeed contribute to the ECM‐integrity by preventing excessive matrix depletion after cartilage trauma.

As NAC treatment already resulted in complete recovery of the cell viability after cartilage trauma, no additive effects were found regarding the combined approach. On the contrary, IGF‐1 largely impaired the NAC‐mediated cell and chondroprotection after trauma, as demonstrated by comparatively low cell viability, enhanced gene expression of ADAMTS‐4, MMP‐1, MMP‐3 and MMP‐13, as well as MMP‐13 secretion and cleavage of type II collagen. That IGF‐1 might lose its pro‐survival and anti‐catabolic attributes in adult human cartilage has already been reported 7. While it is assumed that chondrocyte desensitization towards IGF‐1 increases in correlation to accumulation of IGF‐1 binding proteins as well as ROS and NO, as occurring in elderly or osteoarthritic cartilage tissue 7, 40, efficacy of BMP7 was described as being unaffected by age or OA 18. Nevertheless, single IGF‐1 stimulation was capable to induce type II collagen expression, which indicates that the chondrocytes within the cartilage explants were still susceptible for the pro‐anabolic signalling promoted by IGF‐1 to a certain extent. However, it cannot be completely excluded that the osteoarthritic environment of tissue sampling or the age of the patients might have impaired the pro‐anabolic qualities of IGF‐1 in the context of our study. As NAC is known as an efficient scavenger of harmful ROS/RNS 41, it was expected that the effectiveness of IGF‐1 could be increased in presence of the antioxidant. In fact, a possibly synergistic interaction of IGF‐1 and NAC (2 mM) was initially found regarding COL2A1 expression after trauma, although subsequent analysis of the culture media could not confirm actual enhancement of type II collagen on protein level.

Although the anti‐catabolic qualities of BMP7 18, 19, 20 actually appeared to collaborate with NAC‐mediated suppression of catabolic gene expressions, the combination seemed to induce transcription of ADAMTS‐5. Comparable trends were found for FGF18. Unlike most of the other investigated collagenases and aggrecanases, ADAMTS‐5 is not induced by redox‐sensitive pathways but might be triggered by mechanical stress and subsequent activation of the runt‐related transcription factor (RUNX)‐2 42, 43. Therefore, it remains unclear how NAC or the growth factors might have contributed to the enhanced gene expression of ADAMTS‐5. Along with MMP‐13, ADAMTS‐5 is dealt as one of the most crucial ECM‐degrading enzyme associated with OA pathogenesis 5. As it is suggested that NAC might act as a direct inhibitor of released MMP‐3 in a post‐translational manner, the presence of NAC might prevent ADAMTS‐5 activation by MMP‐3 14, 44.

Regarding NAC‐induced gene expression of type X collagen after trauma, the underlying mechanisms remain largely unclear. Although type X collagen and MMP‐13 are both common biomarkers of hypertrophic chondrocytes, their expression is modulated by various transcriptional regulators and might occur uncoupled 45. This could explain why NAC exhibited divergent influence on the expression of the corresponding genes. Moreover, previous findings indicated that type X collagen is mainly associated with the early stages of OA and was hardly present in later stages while MMP‐13 remained elevated 46. Brew *et al. suggested* that COL10A1‐expressing chondrocytes undergo apoptosis, which might have been prevented by the anti‐apoptotic effects of BMP7, FGF18 and NAC in our study. Another interesting interaction of NAC and BMP7 was shown for the gene expression levels of BMPR1A and 2, which were both enhanced in a synergistic manner, after combined application. Whether and to what degree this might contribute to increased mRNA levels of ADAMTS‐5, COL10A1 or ACAN and other additive effects observed throughout this study should be addressed in future studies.

Although exemplary immunohistochemical staining did not reveal detrimental effects of NAC‐mediated suppression of type II collagen expression on the cartilage characteristics, neither alone nor in combination with BMP7, we are cautiously optimistic as the validity might be limited by the low number of donors. As a consequence of the results we found for the parallel multidirectional approach, future studies may consider a sequential application of the therapeutics, to circumvent the adverse effects observed during simultaneous treatment. Thus, the advantages of both pro‐anabolic and antioxidative therapy could be harnessed. As NAC has been shown to potently attenuate early trauma‐induced processes, it might be advisable to initiate the therapy with the antioxidative approach. To achieve optimal efficacy of NAC, it may be administered for about 7 days 14, 15. After primary harm reduction by NAC, pro‐anabolic stimulation of the surviving chondrocytes towards ECM‐regeneration may follow. Previous findings suggest that therapeutic effects on cell viability and suppression of MMP‐13 secretion might be sustained for at least another week after deprivation of NAC 14. Nevertheless, our data indicate that this does not apply to the inhibition of gene expression and biosynthesis of type II collagen, which in fact exhibited significant recovery after deprivation of NAC. Likewise, NAC‐induced gene expression of COL10A1 has been vanished after withdrawal of the antioxidant. Finally, the effect of growth factor‐based chondroanabolic treatment in a sequential setting after antioxidative therapy and its optimal timing is not known at present and clearly deserves further investigation.

## Conflict of interest

The authors confirm that there are no conflicts of interest.

## Supporting information


**Data S1**: Supplementary information.
**S1**: Culture media.
**S2**: Analytical procedure of Live/Dead Staining.
**S3A–C**: Effects of NAC on type II and X expression after deprivation.
**S4A–D**: Gene expression levels of corresponding growth factor receptors.
**S5A–C**: Histological and immunohistochemical analysis after 14d.

## References

[1] Hunter DJ , Schofield D , Callander E . The individual and socioeconomic impact of osteoarthritis. Nat Rev Rheumatol. 2014; 10: 437–41. 24662640 10.1038/nrrheum.2014.44

[2] Pascual‐Garrido C , Chubinskaya S . Potential targets for pharmacologic therapies for prevention of PTA In: Olson MDAS, Guilak PF, editors. Post‐Traumatic Arthritis: pathogenesis, Diagnosis and Management. Boston, MA: Springer US; 2015 pp. 331–42.

[3] Pascual‐Garrido C , Hakimiyan AA , Rappoport L , *et al* Anti‐apoptotic treatments prevent cartilage degradation after acute trauma to human ankle cartilage. Osteoarthritis Cartilage. 2009; 17: 1244–51. 19332178 10.1016/j.joca.2009.03.007 PMC2786219

[4] Chen CT , Burton‐Wurster N , Borden C , *et al* Chondrocyte necrosis and apoptosis in impact damaged articular cartilage. J Orthop Res. 2001; 19: 703–11. 11518282 10.1016/S0736-0266(00)00066-8

[5] Zhang W , Ouyang H , Dass CR , *et al* Current research on pharmacologic and regenerative therapies for osteoarthritis. Bone Res. 2016; 4: 15040. 26962464 10.1038/boneres.2015.40 PMC4772471

[6] Troeberg L , Nagase H . Proteases involved in cartilage matrix degradation in osteoarthritis. Bba‐Proteins Proteom. 2012; 1824: 133–45. 10.1016/j.bbapap.2011.06.020 PMC3219800 21777704

[7] Li Y , Wang Y , Chubinskaya S , *et al* Effects of insulin‐like growth factor‐1 and dexamethasone on cytokine‐challenged cartilage: relevance to post‐traumatic osteoarthritis. Osteoarthritis Cartilage. 2015; 23: 266–74. 25450855 10.1016/j.joca.2014.11.006 PMC4304966

[8] Loeser RF , Gandhi U , Long DL , *et al* Aging and oxidative stress reduce the response of human articular chondrocytes to insulin‐like growth factor 1 and osteogenic protein 1. Arthritis Rheum. 2014; 66: 2201–9. 10.1002/art.38641 PMC4116467 24664641

[9] Ziskoven C , Jäger M , Zilkens C , *et al* Oxidative stress in secondary osteoarthritis: from cartilage destruction to clinical presentation? Orthop Rev. 2010; 2: e23. 10.4081/or.2010.e23 PMC3143971 21808712

[10] Zuscik MJ , Hilton MJ , Zhang XP , *et al* Regulation of chondrogenesis and chondrocyte differentiation by stress. J Clin Invest. 2008; 118: 429–38. 18246193 10.1172/JCI34174 PMC2214711

[11] Chubinskaya S , Kumar B , Merrihew C , *et al* Age‐related changes in cartilage endogenous osteogenic protein‐1 (OP‐1). Biochim Biophys Acta. 2002; 1588: 126–34. 12385776 10.1016/s0925-4439(02)00158-8

[12] Merrihew C , Kumar B , Heretis K , *et al* Alterations in endogenous osteogenic protein‐1 with degeneration of human articular cartilage. J Orthop Res. 2003; 21: 899–907. 12919879 10.1016/S0736-0266(03)00055-X

[13] Akkiraju H , Nohe A . Role of Chondrocytes in Cartilage Formation, Progression of Osteoarthritis and Cartilage Regeneration. J Dev Biol. 2015; 3: 177–92. 27347486 10.3390/jdb3040177 PMC4916494

[14] Riegger J , Joos H , Palm HG , *et al* Antioxidative therapy in an *ex vivo* human cartilage trauma‐model: attenuation of trauma‐induced cell loss and ECM‐destructive enzymes by N‐acetyl cysteine. Osteoarthritis Cartilage. 2016; 24: 2171–80. 27514995 10.1016/j.joca.2016.07.019

[15] Beecher BR , Martin JA , Pedersen DR , *et al* Antioxidants block cyclic loading induced chondrocyte death. Iowa Orthop J. 2007; 27: 1–8. 17907423 PMC2150661

[16] Hui W , Rowan AD , Cawston T . Insulin‐like growth factor 1 blocks collagen release and down regulates matrix metalloproteinase‐1, ‐3, ‐8, and ‐13 mRNA expression in bovine nasal cartilage stimulated with oncostatin M in combination with interleukin 1alpha. Ann Rheum Dis. 2001; 60: 254–61. 11171688 10.1136/ard.60.3.254 PMC1753584

[17] Seifarth C , Csaki C , Shakibaei M . Anabolic actions of IGF‐I and TGF‐beta1 on Interleukin‐1beta‐treated human articular chondrocytes: evaluation in two and three dimensional cultures. Histol Histopathol. 2009; 24: 1245–62. 19688693 10.14670/HH-24.1245

[18] Chubinskaya S , Hurtig M , Rueger DC . OP‐1/BMP‐7 in cartilage repair. Int Orthop. 2007; 31: 773–81. 17687553 10.1007/s00264-007-0423-9 PMC2266666

[19] Badlani N , Inoue A , Healey R , *et al* The protective effect of OP‐1 on articular cartilage in the development of osteoarthritis. Osteoarthritis Cartilage. 2008; 16: 600–6. 17977753 10.1016/j.joca.2007.09.009

[20] Caron MM , Emans PJ , Cremers A , *et al* Hypertrophic differentiation during chondrogenic differentiation of progenitor cells is stimulated by BMP‐2 but suppressed by BMP‐7. Osteoarthritis Cartilage. 2013; 21: 604–13. 23353668 10.1016/j.joca.2013.01.009

[21] Barr L , Getgood A , Guehring H , *et al* The effect of recombinant human fibroblast growth factor‐18 on articular cartilage following single impact load. J Orthop Res. 2014; 32: 923–7. 24719286 10.1002/jor.22622

[22] Moore EE , Bendele AM , Thompson DL , *et al* Fibroblast growth factor‐18 stimulates chondrogenesis and cartilage repair in a rat model of injury‐induced osteoarthritis. Osteoarthritis Cartilage. 2005; 13: 623–31. 15896984 10.1016/j.joca.2005.03.003

[23] Ellsworth JL , Berry J , Bukowski T , *et al* Fibroblast growth factor‐18 is a trophic factor for mature chondrocytes and their progenitors. Osteoarthritis Cartilage. 2002; 10: 308–20. 11950254 10.1053/joca.2002.0514

[24] Lotz M , Martel‐Pelletier J , Christiansen C , *et al* Republished: value of biomarkers in osteoarthritis: current status and perspectives. Postgrad Med J. 1061; 2014: 171–8. 10.1136/postgradmedj-2013-203726rep PMC3934547 24534711

[25] Kleemann RU , Krocker D , Cedraro A , *et al* Altered cartilage mechanics and histology in knee osteoarthritis: relation to clinical assessment (ICRS Grade). Osteoarthritis Cartilage. 2005; 13: 958–63. 16139530 10.1016/j.joca.2005.06.008

[26] Joos H , Hogrefe C , Rieger L , *et al* Single impact trauma in human early‐stage osteoarthritic cartilage: implication of prostaglandin D2 but no additive effect of IL‐1beta on cell survival. Int J Mol Med. 2011; 28: 271–7. 21567074 10.3892/ijmm.2011.694

[27] Lotz MK , Kraus VB . New developments in osteoarthritis. Posttraumatic osteoarthritis: pathogenesis and pharmacological treatment options. Arthritis Res Ther. 2010; 12: 211. 20602810 10.1186/ar3046 PMC2911903

[28] Chubinskaya S , Wimmer MA . Key Pathways to Prevent Posttraumatic Arthritis for Future Molecule‐Based Therapy. Cartilage. 2013; 4: 13S–21S. 26069661 10.1177/1947603513487457 PMC4297064

[29] Im HJ , Pacione C , Chubinskaya S , *et al* Inhibitory effects of insulin‐like growth factor‐1 and osteogenic protein‐1 on fibronectin fragment‐ and interleukin‐1beta‐stimulated matrix metalloproteinase‐13 expression in human chondrocytes. J Biol Chem. 2003; 278: 25386–94. 12734180 10.1074/jbc.M302048200 PMC2895259

[30] Zhang M , Zhou Q , Liang QQ , *et al* IGF‐1 regulation of type II collagen and MMP‐13 expression in rat endplate chondrocytes *via* distinct signaling pathways. Osteoarthritis Cartilage. 2009; 17: 100–6. 18595745 10.1016/j.joca.2008.05.007

[31] Lotz MK , Otsuki S , Grogan SP , *et al* Cartilage cell clusters. Arthritis Rheum. 2010; 62: 2206–18. 20506158 10.1002/art.27528 PMC2921934

[32] Higgins TF , Johnson BD . Effect of exogenous IGF‐1 on chondrocyte apoptosis in a rabbit intraarticular osteotomy model. J Orthop Res. 2010; 28: 125–30. 19585592 10.1002/jor.20942

[33] Guntur AR , Rosen CJ . IGF‐1 regulation of key signaling pathways in bone. Bonekey Rep. 2013; 2: 437. 24422135 10.1038/bonekey.2013.171 PMC3818534

[34] Knauper V , Will H , Lopez‐Otin C , *et al* Cellular mechanisms for human procollagenase‐3 (MMP‐13) activation. Evidence that MT1‐MMP (MMP‐14) and gelatinase a (MMP‐2) are able to generate active enzyme. J Biol Chem. 1996; 271: 17124–31. 8663255 10.1074/jbc.271.29.17124

[35] Yamaoka H , Nishizawa S , Asawa Y , *et al* Involvement of fibroblast growth factor 18 in dedifferentiation of cultured human chondrocytes. Cell Prolif. 2010; 43: 67–76. 19909293 10.1111/j.1365-2184.2009.00655.x PMC6496437

[36] Shu C , Smith SM , Little CB , *et al* Use of FGF‐2 and FGF‐18 to direct bone marrow stromal stem cells to chondrogenic and osteogenic lineages. Future Sci OA. 2016; 2: FSO142. 10.4155/fsoa-2016-0034 PMC5242207 28116125

[37] Ohbayashi N , Shibayama M , Kurotaki Y , *et al* FGF18 is required for normal cell proliferation and differentiation during osteogenesis and chondrogenesis. Gene Dev. 2002; 16: 870–9. 11937494 10.1101/gad.965702 PMC186331

[38] Davidson D , Blanc A , Filion D , *et al* Fibroblast growth factor (FGF) 18 signals through FGF receptor 3 to promote chondrogenesis. J Biol Chem. 2005; 280: 20509–15. 15781473 10.1074/jbc.M410148200

[39] Yan D , Chen D , Cool SM , *et al* Fibroblast growth factor receptor 1 is principally responsible for fibroblast growth factor 2‐induced catabolic activities in human articular chondrocytes. Arthritis Res Ther. 2011; 13: doi: 10.1186/ar3441. 10.1186/ar3441 PMC3239372 21835001

[40] Loeser RF , Carlson CS , Del Carlo M , *et al* Detection of nitrotyrosine in aging and osteoarthritic cartilage: correlation of oxidative damage with the presence of interleukin‐1beta and with chondrocyte resistance to insulin‐like growth factor 1. Arthritis Rheum. 2002; 46: 2349–57. 12355482 10.1002/art.10496

[41] Aruoma OI , Halliwell B , Hoey BM , *et al* The antioxidant action of N‐acetylcysteine: its reaction with hydrogen peroxide, hydroxyl radical, superoxide, and hypochlorous acid. Free Radic Biol Med. 1989; 6: 593–7. 2546864 10.1016/0891-5849(89)90066-x

[42] Tetsunaga T , Nishida K , Furumatsu T , *et al* Regulation of mechanical stress‐induced MMP‐13 and ADAMTS‐5 expression by RUNX‐2 transcriptional factor in SW1353 chondrocyte‐like cells. Osteoarthritis Cartilage. 2011; 19: 222–32. 21094261 10.1016/j.joca.2010.11.004

[43] Bondeson J , Wainwright S , Hughes C , *et al* The regulation of the ADAMTS4 and ADAMTS5 aggrecanases in osteoarthritis: a review. Clin Exp Rheumatol. 2008; 26: 139–45. 18328163

[44] Echtermeyer F , Bertrand J , Dreier R , *et al* Syndecan‐4 regulates ADAMTS‐5 activation and cartilage breakdown in osteoarthritis. Nat Med. 2009; 15: 1072–6. 19684582 10.1038/nm.1998

[45] van der Kraan PM , van den Berg WB . Chondrocyte hypertrophy and osteoarthritis: role in initiation and progression of cartilage degeneration? Osteoarthritis Cartilage. 2012; 20: 223–32. 22178514 10.1016/j.joca.2011.12.003

[46] Brew CJ , Clegg PD , Boot‐Handford RP , *et al* Gene expression in human chondrocytes in late osteoarthritis is changed in both fibrillated and intact cartilage without evidence of generalised chondrocyte hypertrophy. Ann Rheum Dis. 2010; 69: 234–40. 19103633 10.1136/ard.2008.097139

