# Supplementary material for: Striking a new path in reducing cartilage breakdown: combination of antioxidative therapy and chondroanabolic stimulation after blunt cartilage trauma
Source: J Cell Mol Med. 2017 Aug 22;22(1):77–88. doi: 10.1111/jcmm.13295 (PMC5742720; doi:10.1111/jcmm.13295)
Supplement: Supplementary file 1 — Data S1: Supplementary information. S1: Culture media. S2: Analytical procedure of Live/Dead Staining. S3A–C: Effects of NAC on type II and X expression after deprivation. S4A–D: Gene expression levels of corresponding growth factor receptors. S5A–C: Histological and immunohistochemical analysis after 14d. [file JCMM-22-77-s001.docx]

**Data S1**

**emonstratedore andus remain ng for an ideal timeregimen. to find first f matrix-destructive proteases in both, impacted cartilSupplementary material**

**S1: Culture media.**

Serum-containing medium: 1:1 DMEM/Ham’s F12 supplemented with 10% fetal bovine serum, 0.5% penicillin/ streptomycin (PAA Laboratories, Pasching, Austria), 0.5% L-glutamine and 10 μg/ml 2-phospho-L-ascorbic acid trisodium salt (Sigma-Aldrich, Fluka, Seelze, Germany).

Serum-free medium: DMEM supplemented with 1% sodium pyruvate, 0.5% L-glutamine, 1% non-essential amino acids, 0.5% penicillin/streptomycin and 0.1% insulin-transferrin-sodium selenite (Sigma-Aldrich). All chemicals were purchased from Biochrom (Berlin, Germany) unless specified otherwise.

**S2: Analytical procedure of Live/Dead Staining.**

Unfixed tissue sections (0.5 mm thickness) were stained as described in the material and methods section. 3 pictures (5-fold magnitude) were made from each tissue section. All cells on the picture were counted manually (Image J software). The average count per picture was about 2,000 cells. Due to the preparation of the cartilage punches, the marginal regions sometimes showed increased cell death. Therefore, the immediate vicinity to these regions was spared. As the calcification of the cell-poor calcified zone occasionally spreads into the deep zone and disturbs the fluorescence staining, these areas were also spared if necessary. All pictures included the cell-rich superficial zone, middle zone and the upper portion of the un-calcified deep zone. In general, the complete cross-section was covered this way. In bovine cartilage, the calcification is empirically less severe, so that control experiments in a comparable bovine trauma-model were used to confirm the findings (data not published yet).

**S3: Effects of NAC on type II collagen expression after deprivation.**


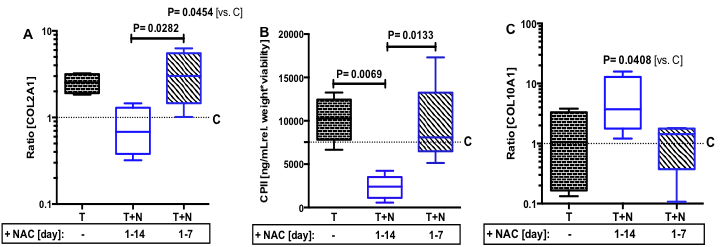


**Fig. S3: NAC-mediated suppression of type II collagen synthesis and induction of COL10A1 gene expression were ceased after deprivation of the antioxidant.** Impacted human cartilage explants were treated either continuously (14d) or for 7d with NAC. Gene expression of COL2A1 **(A)** and COL10A1 (C), respectively, as well as release of CPII **(B**) were evaluated 14d post trauma by qRT-PCR and ELISA, respectively. T= traumatized, +N= treated with NAC (2 mM), n=4, each.

**S4: Gene expression levels of corresponding growth factor receptors.**

**A**

**B**

**D**

**C**

**Fig. S4: Gene expression levels of corresponding growth factor receptors were differently influenced by trauma, growth factor stimulation and NAC treatment, respectively.** Human cartilage explants were stimulated by growth factors (IGF-1=I, FGF18=F or BMP7=B) and/ or treated with NAC. Gene expression of BMPR1A **(A)**, BMPR2 **(B)**, IGFR1 **(C)** and FGFR3 **(D)** were evaluated 7d post trauma by qRT-PCR. T= traumatized, +N= treated with NAC (3.5 mM), n=3-5.

**S5: Histological and immunohistochemical analysis of the main cartilage components after 14d.**

To evaluate cell distribution, content of glycosaminoglycans (GAGs) and type II collagen, within the cartilage tissue, appropriate histological and immunohistochemical analysis were performed exemplarily (n=1). Except for hematoxylin and eosin (HE) staining, which includes the analysis of an impacted cartilage explant at an earlier time point (7d post trauma), all explants were cultivated for 14d. In short, **(A)** routine Mayer´s HE staining was performed to visualize cell count and distribution. **(B)** GAGs of human cartilage explants were stained blue with a 1% alizarin blue (Sigma-Aldrich, Taufkirchen, Germany) staining solution (in 3% acetic acid; pH 2.5). **(C)** Type II collagen was detected by immunohistochemical analysis using a specific primary antibody (Acris, Hiddenhausen, Germany; polyclonal) and the Dako LSAB2 System-HRP kit (Dako, Glostrup, Denmark). Negative control (NC) was performed in absence of the primary antibody

**A**


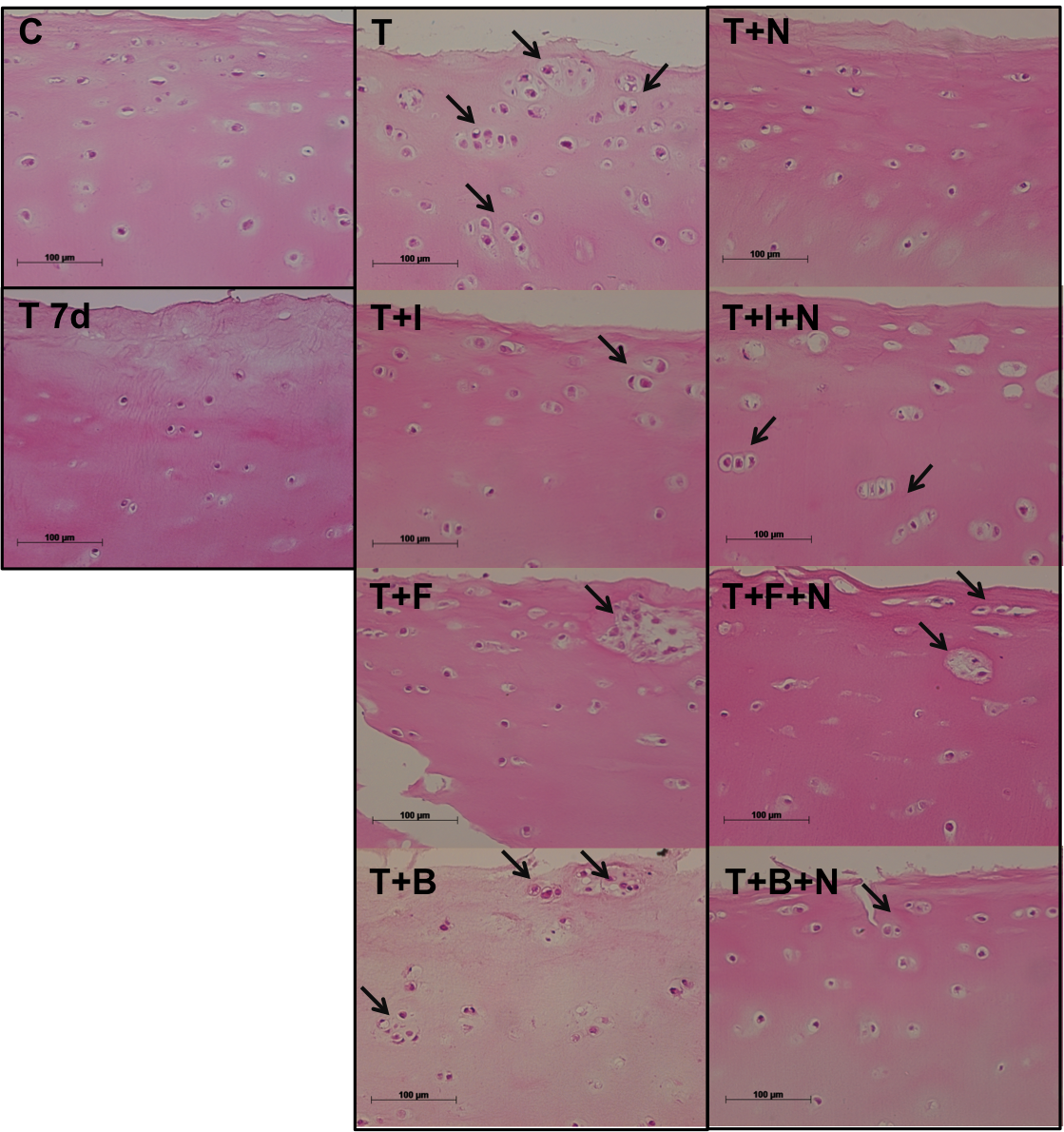


**Fig. S4A: Hematoxylin and eosin (H&E) staining of human cartilage explants.** After trauma (T), human cartilage explants were continuously stimulated by growth factors IGF-1(I), FGF18 (F) or BMP7 (B) and/ or treated with 2 mM NAC (N) for 14d. T 7d = impacted cartilage explant after 7d (earlier time point). Arrows highlight trauma-associated aberration of the cell distribution, including cell clone or cluster formation due to enhanced proliferation and column-like stacking. Impacted cartilage explants showed hypocellularity and less cluster formation at the earlier time point (7d) as compared to the later time pint (14d) after trauma.

**B**


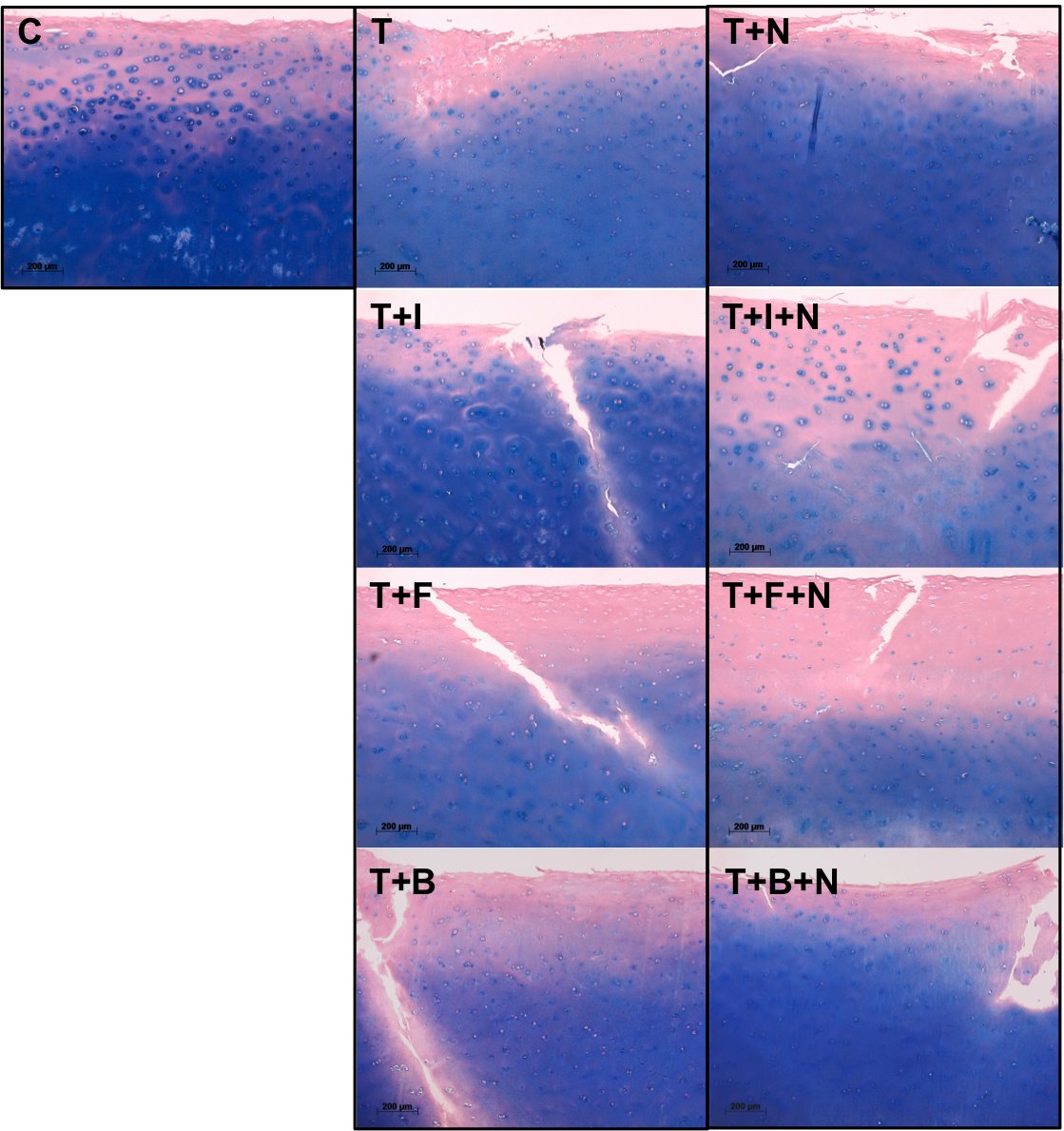


**Fig. S3B: Alizarin blue staining of human cartilage explants.** After trauma (T), human cartilage explants were continuously stimulated by growth factors IGF-1(I), FGF18 (F) or BMP7 (B) and/ or treated with 2 mM NAC (N) for 14d.

**
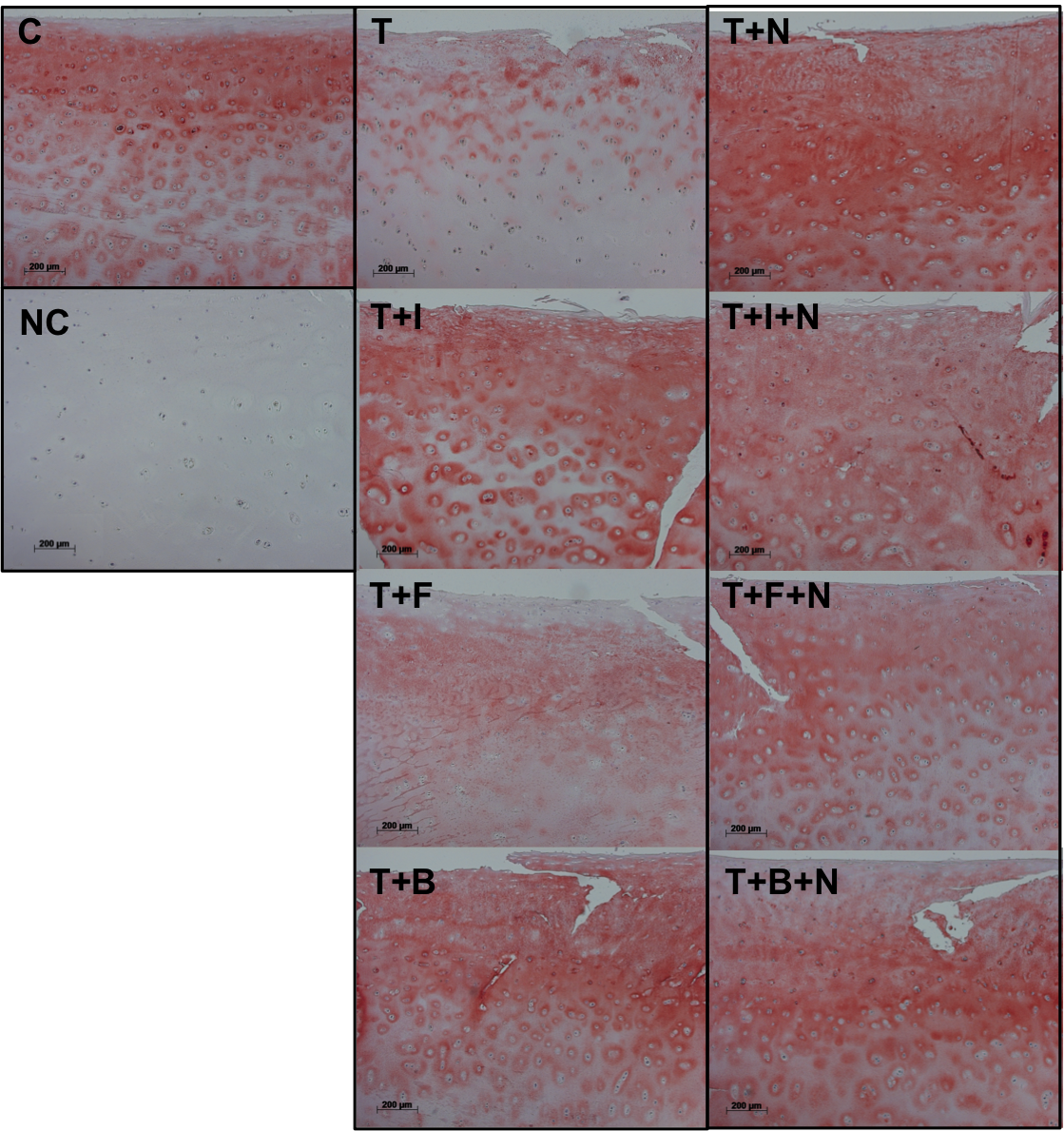
**

**Fig. S4C: Type II collagen immunohistological staining of human cartilage explants.** After trauma (T), human cartilage explants were continuously stimulated by growth factors IGF-1 (I), FGF18 (F) or BMP7 (B) and/ or treated with 2 mM NAC (N) for 14d. NC = negative control.
